# Medical, dental, and nursing students’ attitudes and knowledge towards artificial intelligence: a systematic review and meta-analysis

**DOI:** 10.1186/s12909-024-05406-1

**Published:** 2024-04-15

**Authors:** Hamidreza Amiri, Samira Peiravi, Seyedeh sara rezazadeh shojaee, Motahareh Rouhparvarzamin, Mohammad Naser Nateghi, Mohammad Hossein Etemadi, Mahdie ShojaeiBaghini, Farhan Musaie, Mohammad Hossein Anvari, Mahsa Asadi Anar

**Affiliations:** 1 Student Research Committee, Arak University of Medical Sciences, Arak, Iran; 2 Department of Emergency Medicine, Faculty of Medicine, Mashhad University of Medical Sciences, Mashhad, Iran; 3 Department of Nursing, Faculty of Nursing and Midwifery, Mashhad Medical Sciences, Islamic Azad University, Mashhad, Iran; 4 Student Research Committee, School of Nursing and Midwifery, Shahid Sadoughi University of Medical Sciences, Yazd, Iran; 5 Student Research Committee, Faculty of Nursing and Midwifery, Mashhad University of Medical Sciences, Mashhad, Iran; 6 Students Research Committee, School of Medicine, Isfahan University of Medical Sciences, Isfahan, Iran; 7 Medical Informatics Research Center, Institute for Futures Studies in Health, Kerman University of Medical Sciences, Kerman, Iran; 8 Dentistry Student, Dental Branch, Islamic Azad University, Tehran, Iran; 9 Master of Health Science, Faculty of Health Sciences, Universiti Kebangsaan Malaysia (UKM), Kuala Lumpur, Malaysia; 10 Student Research Committee, School of Medicine, Shahid Beheshti University of Medical Sciences, SBUMS, Arabi Ave, Daneshjoo Blvd, Velenjak, Tehran, 19839-63113 Iran

**Keywords:** Artificial intelligence, AI, Medical students, Dental students, Nursing students, Meta-analysis, Systematic review

## Abstract

**Background:**

Nowadays, Artificial intelligence (AI) is one of the most popular topics that can be integrated into healthcare activities. Currently, AI is used in specialized fields such as radiology, pathology, and ophthalmology. Despite the advantages of AI, the fear of human labor being replaced by this technology makes some students reluctant to choose specific fields. This meta-analysis aims to investigate the knowledge and attitude of medical, dental, and nursing students and experts in this field about AI and its application.

**Method:**

This study was designed based on PRISMA guidelines. PubMed, Scopus, and Google Scholar databases were searched with relevant keywords. After study selection according to inclusion criteria, data of knowledge and attitude were extracted for meta-analysis.

**Result:**

Twenty-two studies included 8491 participants were included in this meta-analysis. The pooled analysis revealed a proportion of 0.44 (95%CI = [0.34, 0.54], *P* < 0.01, I2 = 98.95%) for knowledge. Moreover, the proportion of attitude was 0.65 (95%CI = [0.55, 0.75], *P* < 0.01, I2 = 99.47%). The studies did not show any publication bias with a symmetrical funnel plot.

**Conclusion:**

Average levels of knowledge indicate the necessity of including relevant educational programs in the student’s academic curriculum. The positive attitude of students promises the acceptance of AI technology. However, dealing with ethics education in AI and the aspects of human-AI cooperation are discussed. Future longitudinal studies could follow students to provide more data to guide how AI can be incorporated into education.

**Supplementary Information:**

The online version contains supplementary material available at 10.1186/s12909-024-05406-1.

## Introduction

The term "artificial intelligence (AI)" was coined nearly 70 years ago to refer to using of computers to imitate human reasoning [1]. The first application of AI was in mathematics in 1956 when it was utilized for proving theorems [2]. Integrating of AI in medicine was a gradual process [3] that began with the development of a software program that guided doctors on appropriate antimicrobial therapy [4].

AI is a trending topic that is currently at the forefront of technological advancements [5] and has the potential to influence the healthcare industry significantly [6]. The term AI refers to a scientific and engineering discipline that deals with developing computer-based systems capable of exhibiting intelligent behavior, as well as understanding and replicating human-like cognitive processes [7]. Recent advancements in computer and informatics technologies have paved the way for integrating of AI technologies, such as machine learning and deep learning, into healthcare information systems [8, 9]. AI has been extensively integrated into decision support systems (DSSs) in data-intensive medical specialties like radiology, pathology, and ophthalmology [10].

Several experts have expressed their opinions on the future of radiology in light of AI's emergence [11, 12]. Radiological societies have also published white papers promoting their views [13, 14]. Studies have indicated that medical students do not express significant concern or fear about being replaced by AI in their profession [15]. However, some students may experience anxiety related to the possibility of being displaced by AI, which may discourage them from considering certain medical specialties [16]. Indeed, there are positive and negative perspectives on the impact of AI on daily human life. Pessimistic views suggest that AI may replace humans in various sectors. On the other hand, optimistic views highlight that individuals with AI support will have increased opportunities to leverage future advancements [17]. To the best of our knowledge, this study aimed to evaluate the attitudes, knowledge, and skills of medical, dental, and nursing students toward AI and to gather information about their opinions on the use of AI.

## Method

This systematic review and meta-analysis study was based on the PRISMA (Preferred Reporting Items for Systematic Reviews and Meta-Analysis) guidelines. The protocol of this study was registered on PROSPERO with the ID of *CRD42024521006.*

### Literature search

A structured literature search was applied up to 12th September 2023 to collect appropriate articles from PubMed /MEDLINE, Scopus, and Google Scholar databases. Search tactics included two main subgroups of keywords. One subgroup was the concepts related to artificial intelligence, and the other group was the perspective of health care and dentists; then, Subgroups were mixed by using ‘AND.’ More specifically, we searched the above databases for (artificial intelligence or machine learning) and (Medical or dentistry or nursing) (Table 1). The search process was done according to the query options of each database. In addition, we searched the reference lists of appropriate systematic reviews to prevent missing data. Two reviewers accomplished all strategies in a solitary state, and any controversy between the reviewers was resolved by negotiation.

**Table 1:** Search strategy of the current study for online databased including PubMed and Scopus

Search Engines	Search Strategy	Additional Filters / Date
PubMed/Medline	(Medical[ti] OR dental[ti] OR dentistry[ti] OR nursing[ti] OR healthcare[ti]) AND (artificial intelligence[ti] OR machine learning[ti] OR AI[ti])	None / 12th September 2023
Scopus	TITLE(Medical OR dental OR dentistry OR nursing OR healthcare) AND TITLE(artificial intelligence OR machine learning OR AI)	None / 12th September 2023
Google scholar	allintitle: (Medical OR dental OR dentistry OR nursing OR healthcare) AND (artificial intelligence OR machine learning OR AI)	None / 12th September 2023

### Criteria for selecting studies

The main goal was to evaluate the attitudes of students and graduates working in dentistry, nursing, or medical (health care providers) fields toward AI and machine learning. We didn't use any restrictions on date and language, but to make the search more specific, we restricted the keyword search to the title. Articles with irrelevant subject matter and studies utilizing animal models were excluded during the initial phase of document selection. Additionally, duplicate documents were eliminated.

### Data extraction and study quality assessment

Two reviewers independently assessed the title and abstract of each study to ascertain its suitability for inclusion in this meta-analysis. We excluded studies that didn’t fulfill our criteria. The complete text of the remaining studies was reviewed, and studies that met the criteria were included in the data extraction step. After that, the subsequent items were acquired for extraction and divided into four sets:

- 1. Study characteristics include authors, type of study, year, location, and follow-up duration.
- 2. Participant variables (average age, gender).
- 3. Research Methodology (e.g., participant sample size).
- 4. Results and outcomes (the attitude, knowledge, and skill toward artificial intelligence).

Two previously mentioned reviewers utilized the critical appraisal checklists for cohort, case–control, and analytical cross-sectional studies created by the Joanna Briggs Institute (JBI). The checklists can be found at the following website: https://jbi.global/critical-appraisal-tools. If there were any inconsistencies, a third author was involved in the process.

### Statistical analysis

Our data analysis was conducted using the STATA 13.1 software developed by StataCorp LP in College Station, TX, USA. The findings were presented as combined odds ratios (ORs) and a 95% confidence interval displayed in a forest plot. Heterogeneity among the eligible studies was assessed using the I2 statistic. The random effects model was employed when significant heterogeneity was observed (I2 > 50%). In addition, we performed a sensitivity analysis by systematically excluding one study at a time and repeating the meta-analysis. This allowed us to guarantee the consistency of our conclusions. To assess the possibility of publication bias, we visually examined the symmetry of the funnel plot and conducted Egger’s regression analysis.

## Result

### Search strategy

**Fig. 1:**
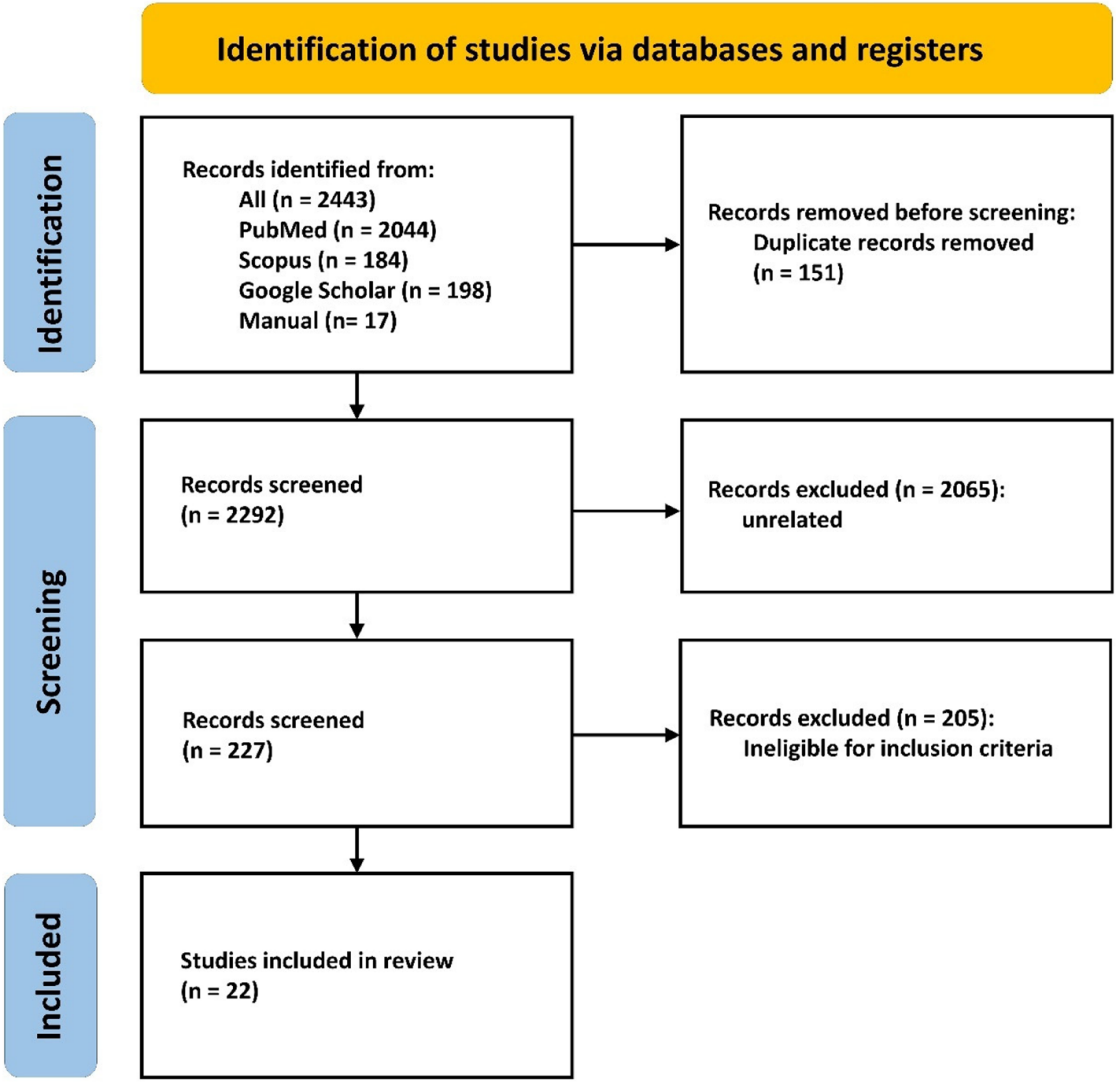
Prisma diagram for study selection process in this study

We obtained 2426 from PubMed/MEDLINE, Scopus, and Google Scholar in the initial search. Seventeen studies were found by manual search. After the automatic removal of duplicated reports, 2292 studies remained. Two thousand sixty-five studies were excluded in the title and abstract evaluation. Two hundred twenty-seven remaining studies underwent additional assessment through full-text, causing 205 papers to be excluded due to ineligibility to inclusion criteria. Finally, 22 studies were included in this systematic review and meta-analysis (Fig. 1).

### Baseline characteristic

This systematic review and meta-analysis evaluated the attitude, knowledge, and skills of medical, dental, and nursing students toward artificial intelligence. We included 22 original articles published from 2020–2023. These studies were performed in several countries, including the U.S.A [18], Germany [19, 20], Lebanon [21], Pakistan [22], Canada [23], The U.K. [24], United Arab Emirates [25], Nigeria [26], Turkey [27, 28], Spain [29] Saudi Arabia [30], India [31–33], Egypt [34], Peru [35], Nepal [36], Kuwait [37], Syria [38], and multiple countries [39]. The study design of 19 studies was cross-sectional [20–38], and the rest followed a mixed methodology [18, 19, 39]. This study included 8491 participants, with a mean age of 19–30 years (Table 2).

**Table 2:** Baseline characteristics of included studies

Author (year) [ref]	Country	Study design	Follow Up Duration	Number of Participants	Mean age	Sex (female)	outcomes
Liu et al. (2022) [52]	The United States	Mixed Methods	-	390 medical students	26 ± 3		Assessment the sight of medical student about involving AI in education
McLennan et al. (2022) [53]	Germany	Cross – sectional	-	844 medical students	19 (median)	545 (66.8%)	Assessment the knowledge and sight of medical student about AI and its usefulness, advantages and disadvantages, role in clinical-decision-making, and issues
Mehta et al. (2021) [23]	Canada	Cross – sectional	-	321 medical students	-	201 (63%)	Assessment the knowledge and perception of medical student about AI and its application in education
Moldt et al. (2023) [19]	Germany	Mixed methods	-	12 medical students	24.8 ± 2.0	6 (50%)	Assessment the knowledge and attitude of medical student about involving AI technology in education
Yüzbaşıoğlu et al. (2020) [27]	Turkey	cross-sectional	1 month	1103 dental students	21.36 ± 1.93	650 (58.9%)	Assessment the attitude and perception of dental student about Ai and its application in dentistry
Hassan Mekawy et al. (2020) [34]	Egypt	cross-sectional	2 months	128 nursing students	21.9 ± 1.7	77 (60.2%)	Assessment the digital health literacy level of nursing student and its relationship with their perception, attitude toward AI utilization
Hamd et al. (2023) [25]	United Arab Emirates	cross-sectional	eight-week	134 including academic staff, clinical dentists, and undergraduate dental students (72)	-	87 (64.9%)	Assessment the knowledge, attitude, and willingness of dentist/dentistry student and their organization to integrate AI
Thulasi et al. (2022) [33]	India	cross-sectional	-	200 dental students and dental practitioners	26.08 ± 4.13	101 (50.5%)	Assessment the knowledge, attitude, and practice of dental student/practitioners toward AI
Swed et al. (2022) [38]	Syria	cross-sectional	36 days	1494 Including 1252 medical students and 255 doctors	25.5	718 (48%)	Predict the knowledge, attitude, and practice of AI taking account baseline characteristic
Ejaz et al. (2022) [39]	48 different countries	Original (mixed-methods)	-	128 medical students	-	72 (56%)	Assessment the perception of medical student about the role of AI in education
Buabbas et al. (2023) [37]	Kuwait	Cross-sectional	-	352 medical students	22.1 ± 1.8 years	313 (88.9%)	Assessment the perception of medical student about role of AI in education
Khanagar et al. (2021) [30]	Saudi Arabia	Cross-sectional	1 month	423 dental students	-	208 (49.2%)	Assessment the knowledge, attitude, and perception of dentistry student toward AI application
Doumat et al. (2022) [21]	Lebanon	Cross-sectional	-	206 medical students	22.7	87 (42.2%)	Assessment the knowledge and attitude of medical student about using AI in education
Ahmed et al. (2022) [22]	Pakistan	cross-sectional	1 month	470 individuals including 223 doctors and 247 medical students	21–30 Median age	231 (49.1%)	Assessment the knowledge, attitude and practice of medical students/doctors about AI
Asmatahasin et al. (2021) [32]	India	cross-sectional	-	270 dental students	24.6 ± 3.03	221 (81.85%)	Assessment the attitude and perception of dental student toward AI utilization in dentistry
Gaye Keser (2021) [28]	Turkey	cross-sectional	-	140 (including 75, 4th and 65, 5th grades dental students)	22.91 ± 1.48	85 (60.7%)	Assessment the knowledge, attitude, and perception of dental student toward AI usage in radiological diagnoses
Nisha Jha (2021) [36]	Nepal	cross-sectional	24 days	216 Medical Students	-	125 (57.9%)	Assessment the knowledge and perception of medical students toward AI and their preference of including AI teaching in study programm
Rohin Kansal (2022) [31]	India	Cross-Sectional	one week	212 Medical Students	-	86 (40.6%)	Assessment the knowledge of medical students/doctors about basic principle, limitation, and application of AI
Milan Karan-Romero (2023) [35]	Peru	cross-sectional	About one year	200 dental students	22 ± 2.87	98 (49%)	Assessment the attitudes and perceptions of dentistry student toward AI usage
Andrés Barreiro-Ares (2323) [29]	Spain	cross-sectional	from 3 January to 31 March 2022	283 Medical Students	(22.2 ± 3.5)	200 (71.17%)	Assessment the perception of undergraduate medical student about current AI situation in medicine and radiology
Sit (2020) et al	The U.K	Cross-sectional		484 medical students	-	-	Assessment the attitude of medical student about AI and its role in radiology
Dere et.al (2023)	Nigeria	Cross-sectional	6 weeks	481 medical students and doctors	-	-	Assessment the knowledge and attitude of the medical students / doctors about AI

### Attitude

**Fig. 2:**
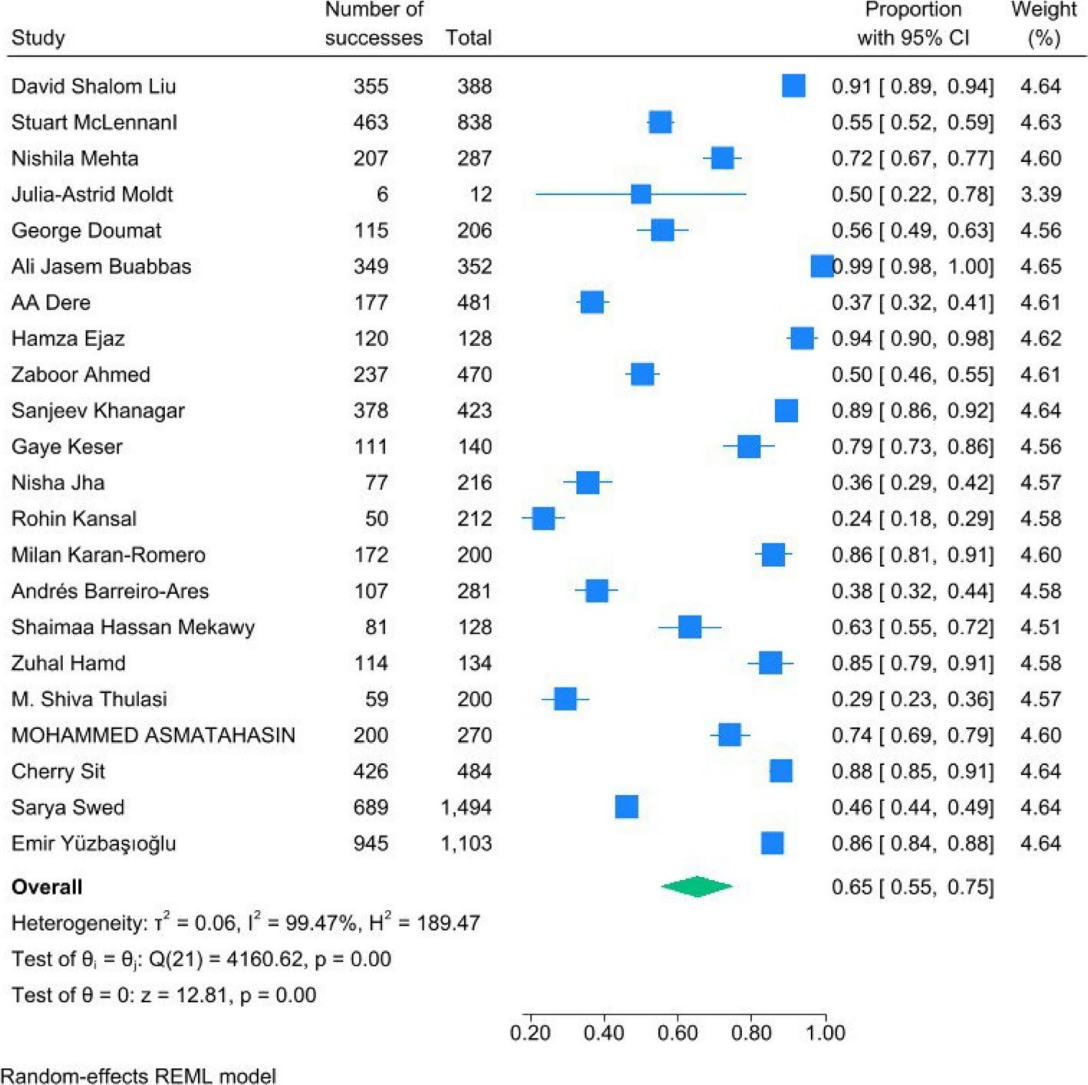
Forest plot of proportion of attitude showed a significant effect of 0.44 (0.34, 0.54)

We performed a meta-analysis on 22 studies for attitude of students toward AI. The proportion for attitude was 0.65 (95%CI = [0.55, 0.75], *P* < 0.01) according to 22 studies. This means that 65% of all students were agree with the use of AI in medicine and had a favorable view. Similarly, the heterogeneity was severe with I2 of 99.47%, and H2 = 189.47 (Fig. 2).

**Fig. 3:**
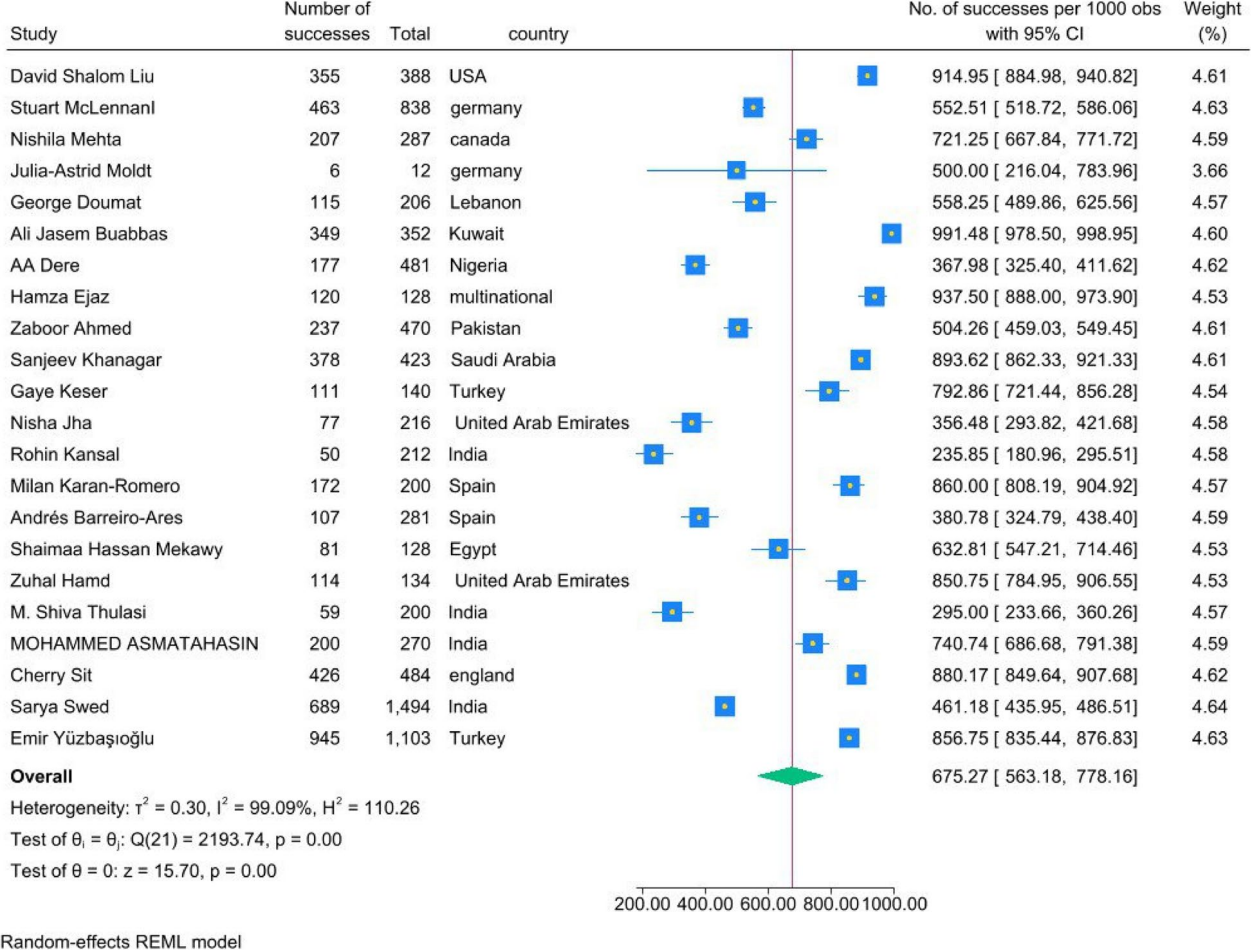
Forest plot for comparing countries in terms of their students' attitudes toward AI

In comparison between various countries, students in the U.S.A. Kuwait, Saudi Arabia, Turkey, and England showed a higher rate of attitude toward AI than those in Germany, Lebanon, Nigeria, Pakistan, and India. Additionally, the Attitudes of Spanish and United Arab Emirates students varied in different studies. Finally, students in Canada and Egypt displayed a medium rate of positive attitude (Fig. 3).

### Knowledge

**Fig. 4:**
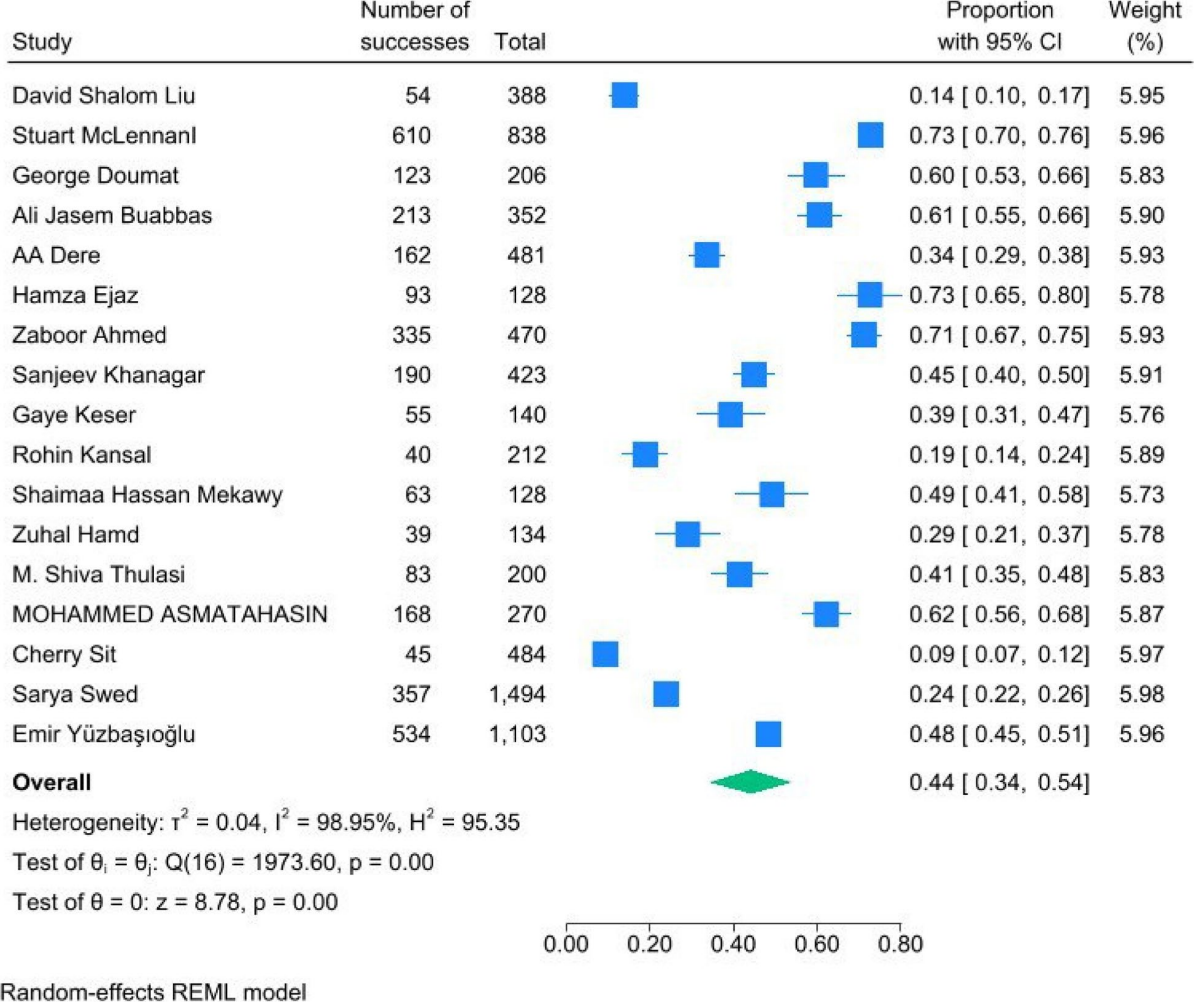
Forest plot of proportion of knowledge showed a significant effect of 0.65 (0.55, 0.75)

A total of 17 studies had provided the knowledge data. The pooled analysis showed a proportion for knowledge of 0.44 (95%CI = [0.34, 0.54], *P* < 0.01). This shows that 44% of the total population of included students had a relatively good knowledge about AI, either in the field of theory or practical. The studies showed a high heterogeneity with an I2 of 98.95% and H2 of 93.35 (Fig. 4).

**Fig. 5:**
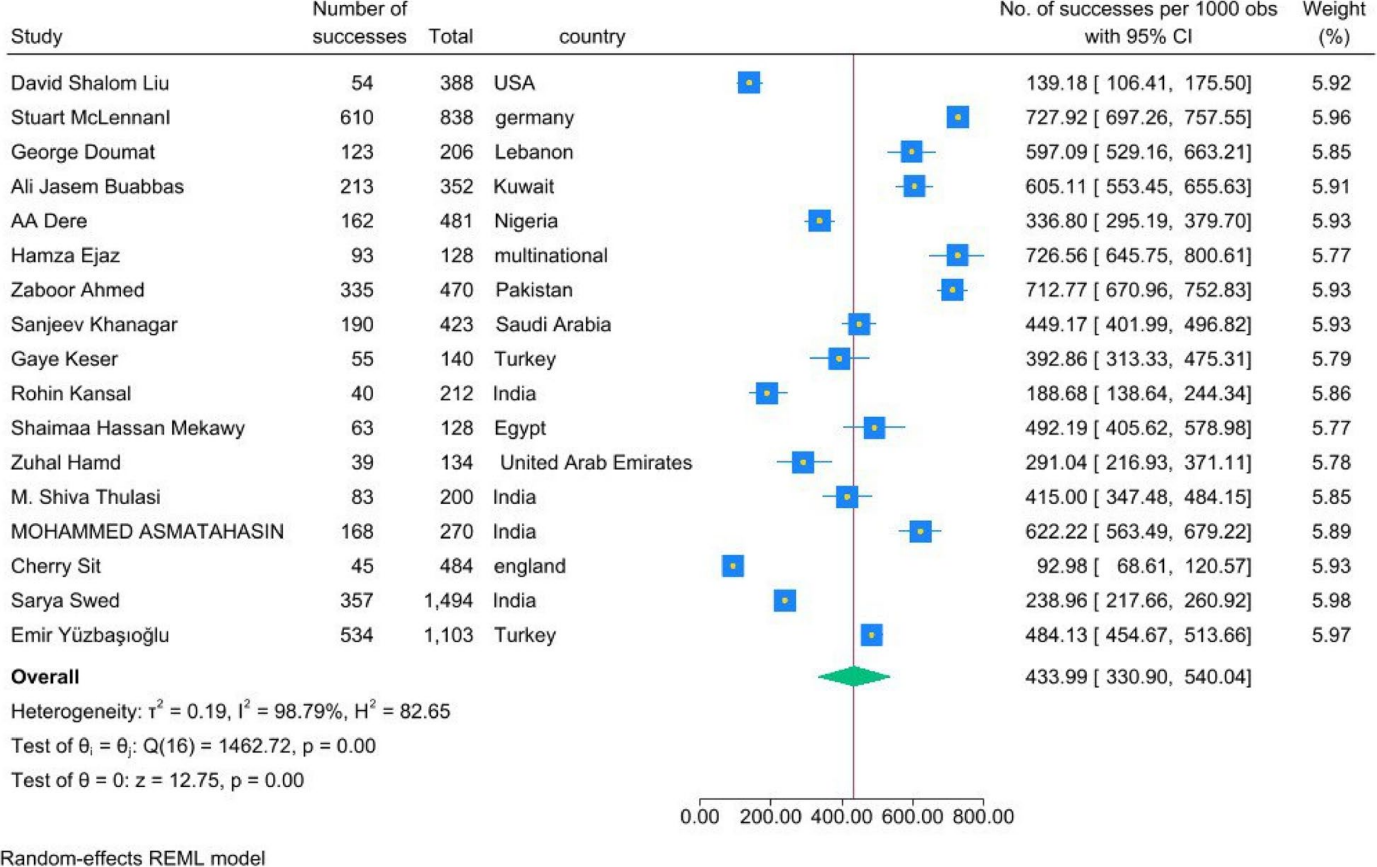
Forest plot for comparing countries in terms of their students' knowledge of AI

Students from Germany, Lebanon, Kuwait, and Pakistan had higher levels of knowledge in the field of AI. In contrast, students from the U.S.A., Nigeria, the United Arab Emirates, and England showed a relatively lower knowledge level. Additionally, the level of knowledge in Indian students varied across different studies. Finally, students from Egypt, Saudi Arabia, and Turkey showed moderate knowledge (Fig. 5).

### Publication bias

**Fig. 6:**
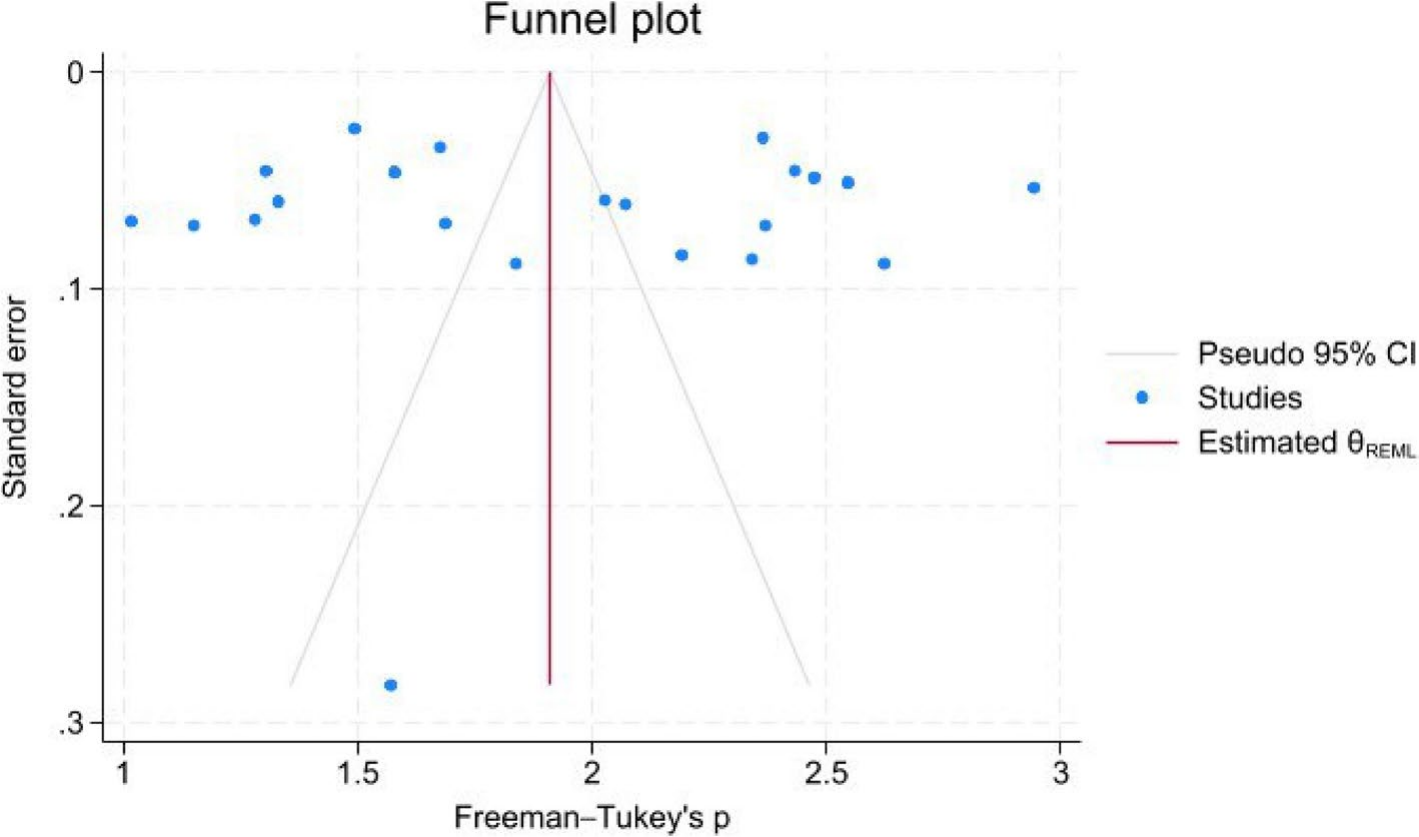
Funnel plot of included studies showed a symmetrical pattern including no publication bias (Egger’s test *P*-value = 0.75)

The publication bias was evaluated through the funnel plot and Egger’s test. The funnel plot (Fig. 6) showed a symmetrical pattern, indicating no publication bias. This was supported by Egger’s test result (*P* = 0.75).

## Discussion

This systematic review and meta-analysis aimed to provide evidence on medical, dental, and nursing students’ attitudes, knowledge, and skills regarding AI. Across 24 studies with 5789 participants, students demonstrated moderate knowledge but generally positive attitudes towards AI.

Overall, 44% of students exhibited medium to high knowledge of AI principles and applications. Knowledge encompassed theoretical understanding of AI algorithms, practical abilities to implement AI systems, and programming proficiency. However, the majority of students had limited AI knowledge. This knowledge gap signals an urgent need to incorporate comprehensive AI education into healthcare curricula. Studies show that students support this idea [40, 41]. Curricula should cover foundational concepts like machine learning and neural networks as well as applied skills in utilizing AI tools for tasks like diagnostic imaging interpretation. Hands-on experiential learning with real-world case examples could prove highly effective. Other reason is that lack of knowledge is an important barrier to the use of AI [42]. Notably, students from developed countries demonstrated greater AI knowledge than peers in developing nations. This has been shown in previous studies as well [43]. This discrepancy highlights concerning global digital divides in accessing AI skills training. Targeted investments and capacity building programs are critical to ensuring students worldwide can gain applied AI competencies.

In contrast to their variable knowledge, 65% of students expressed positive attitudes regarding AI utilization in education and clinical practice. This was also showed in previous studies that most of healthcare students have a positive attitude towards AI [19, 44–47]. Students recognized potential benefits of AI for enhancing diagnostic accuracy, improving healthcare access, and relieving clinical workloads. In contrast there are negative perceptions too [44, 48, 49].

Attitudinal measures had substantial heterogeneity, reflecting divergent perceptions across student subgroups. In particular, developing world students held more skeptical views, fearing AI could dehumanize care or render healthcare jobs obsolete. Curricula must address these valid ethical and social concerns through discussions of AI bias, transparency, and impacts on healthcare roles. It should be noted that patient privacy and autonomy, informed consent, transparency, equality and biases are some of major concerns [50]. Refining attitudinal measures with more granular subsets and exploring predictors of AI acceptance would further inform targeted educational initiatives based on students’ specific concerns.

### Enthusiasm and optimism vs. expertise gaps

Overall students showed enthusiasm and optimism about AI's role in medicine, yet the majority lacked substantial expertise and practical abilities in utilizing AI technology. A similar pattern exists in other majors too. A study by Busch et al. involving 387 pharmacy students from 12 countries found that 58% of students held positive attitudes towards AI in medicine, while 63% reported limited general knowledge of AI [51]. Bridging these attitude-knowledge gaps represents a key challenge for AI readiness. Curricula must not only transfer technical knowledge but also address values, ethics, and societal impacts. Education should emphasize AI as a collaborative tool to augment human capabilities rather than replace them. Again, having students directly experience AI’s benefits for care quality could show its potential for enhancing work rather than displacing workers. Additionally, equitable access to AI upskilling is imperative, particularly for students from disadvantaged regions who may have heightened concerns about AI’s risks.

### Strength and limitations

The strength of our study is the review of articles from three large databases, including PubMed, Scopus, and Google Scholar. Also, we used the random effect model to ensure the robustness of the results. Also, our study had some limitations. We included only studies in English. In addition, most of the included studies used their own questionnaires to evaluate the knowledge and approach of the participants toward artificial intelligence. Finally, it is necessary to mention that there were not enough studies to extract the skill results and perform a meta-analysis.

### Future research directions

Future research should investigate the long-term knowledge and attitudinal trajectories of students after graduation. As AI becomes further embedded into real-world practice, how do provider perspectives evolve? Do knowledge gaps persist or does on-the-job exposure improve understanding? How do early attitudinal concerns translate to technology adoption patterns? Longitudinal data tracking cohorts of students into practice could provide pivotal insights to guide continuing education and change management interventions.

Follow-up studies should also assess the durability of AI skills training. Can one-time education produce lasting competencies or is ongoing reinforcement needed? Comparisons of different pedagogical approaches for AI instruction could illuminate best practices as well. And crucially, future work must evaluate links from AI education to concrete improvements in clinical processes and patient outcomes. Demonstrating benefits to care quality represents the strongest incentive for curriculum reform.

## Conclusion

AI is rapidly transforming healthcare and medical education. However, the extent to which healthcare students are prepared for this transformation remains unclear. The moderate knowledge levels indicate substantial room for improvement through curricular enhancement. Hands-on experiential learning focused on applied AI skills shows promise for durably improving competencies. Positive baseline attitudes bode well for acceptance, but targeted education around AI ethics, impacts, and human-AI collaboration will be key to realizing this potential.

Important gaps remain in understanding long-term knowledge retention, optimal pedagogies, impacts of improved education on clinical processes and outcomes, and equitable global access. Follow-up longitudinal studies tracking cohorts of students into practice could offer pivotal data to guide continuing education. Comparisons of instructional approaches may illuminate best practices.

### Supplementary Information

**Supplementary Materials 1.**

## Data Availability

The datasets generated and analyzed during the current study are not publicly available but are available from the corresponding author on reasonable request.

## Abbreviation

AI: Artificial intelligence
